# TRPC4/TRPC5 channels mediate adverse reaction to the cancer cell cytotoxic agent (-)-Englerin A

**DOI:** 10.18632/oncotarget.25659

**Published:** 2018-07-03

**Authors:** Sin Ying Cheung, Matthias Henrot, Mohammad Al-Saad, Matthias Baumann, Heiko Muller, Anke Unger, Hussein N. Rubaiy, Ilka Mathar, Klaus Dinkel, Peter Nussbaumer, Bert Klebl, Marc Freichel, Baptiste Rode, Sebastian Trainor, Steven J. Clapcote, Mathias Christmann, Herbert Waldmann, Syed Khawar Abbas, David J. Beech, Naveen S. Vasudev

**Affiliations:** ^1^ School of Medicine, University of Leeds, Leeds, LS2 9JT, England, UK; ^2^ Institute of Chemistry and Biochemistry, Freie Universität Berlin, 14195 Berlin, Germany; ^3^ School of Biomedical Sciences, University of Leeds, Leeds, LS2 9JT, UK; ^4^ Lead Discovery Center GmbH, D-44227 Dortmund, Germany; ^5^ Institute of Pharmacology, Universität Heidelberg, D-69120 Heidelberg, Germany; ^6^ Max-Planck-Institut für Molekulare Physiologie, D-44227 Dortmund, Germany; ^7^ Technische Universität Dortmund, Fakultät für Chemie und Chemische Biologie, D-44227 Dortmund, Germany

**Keywords:** TRPC4 channels, TRPC5 channels, renal cancer, breast cancer, Ewing’s sarcoma

## Abstract

(-)-Englerin A (EA) is a natural product which has potent cytotoxic effects on renal cell carcinoma cells and other types of cancer cell but not non-cancer cells. Although selectively cytotoxic to cancer cells, adverse reaction in mice and rats has been suggested. EA is a remarkably potent activator of ion channels formed by Transient Receptor Potential Canonical 4 and 5 proteins (TRPC4 and TRPC5) and TRPC4 is essential for EA-mediated cancer cell cytotoxicity. Here we specifically investigated the relevance of TRPC4 and TRPC5 to the adverse reaction. Injection of EA (2 mg.kg^-1^ i.p.) adversely affected mice for about 1 hour, manifesting as a marked reduction in locomotor activity, after which they fully recovered. TRPC4 and TRPC5 single knockout mice were partially protected and double knockout mice fully protected. TRPC4/TRPC5 double knockout mice were also protected against intravenous injection of EA. Importance of TRPC4/TRPC5 channels was further suggested by pre-administration of Compound 31 (Pico145), a potent and selective small-molecule inhibitor of TRPC4/TRPC5 channels which did not cause adverse reaction itself but prevented adverse reaction to EA. EA was detected in the plasma but not the brain and so peripheral mechanisms were implicated but not identified. The data confirm the existence of adverse reaction to EA in mice and suggest that it depends on a combination of TRPC4 and TRPC5 which therefore overlaps partially with TRPC4-dependent cancer cell cytotoxicity. The underlying nature of the observed adverse reaction to EA, as a consequence of TRPC4/TRPC5 channel activation, remains unclear and warrants further investigation.

## INTRODUCTION

Three of the most challenging cancers are renal cell carcinoma, triple negative breast cancer and Ewing’s sarcoma [1–4]. Therefore it has been interesting to see the recent discovery of (-)-Englerin A (EA), a natural sesquiterpene from the *Phyllanthus engleri* plant, as a potent cytotoxic agent against some cancer cell lines developed from patients with these cancers [5–9]. Cancer cell lines from other types of cancer are also killed by EA whereas other cancer cells are resistant, as are non-cancerous cells [6–8]. EA may therefore be a starting point for developing new types of agent which are effective against certain types of cancer for which innovative treatment strategies are urgently needed.

If EA is to be the basis for therapeutic drug discovery, it is important to know the underlying molecular mechanisms by which it achieves cancer cell-specific cytotoxicity. Surprisingly, EA was found to be a remarkably efficacious agonist of the TRPC4 and TRPC5 ion channels [7, 8, 10]. It has nanomolar potency at these channels and is apparently directly-acting and specific [7, 8]. Importantly, the cytotoxicity of EA against cancer cells is strongly TRPC4-dependent [7, 10]. The mechanism of cytotoxicity depends on Na^+^ entry through the TRPC4 channels [10]. Consistent with this mechanism, the effect of EA is potentiated by ouabain, an inhibitor of Na^+^ K^+^-ATPase, suggesting that vulnerability to EA-induced cytotoxicity depends on a combination of excess sustained Na^+^ entry (through TRPC4 channels) and insufficient compensation by Na^+^ extrusion (by Na^+^ K^+^-ATPase) [10]. EA activates TRPC5 channels [8] but it has been suggested that this channel might only rarely be relevant to cancer cell cytotoxicity [7], although the topic is worthy of further investigation because of the suggested importance of TRPC5 in chemotherapy-resistant breast cancer [11]. EA might have other targets and a prominent suggestion in this regard is the protein kinase C, PKCθ [9]. Whether the PKCθ mechanism is related to TRPC4 channels is unknown.

Despite the promising mechanistic findings and lack of effect of EA on non-cancer cells in culture, a challenge with EA might be adverse effect *in vivo*: Although an initial study used EA successfully *in vivo* in mice to inhibit xenograft tumor growth, without notable adverse effect, a subsequent study suggested unacceptable toxicity [7, 9]. A key question in this situation is whether any observed adverse effects of EA represent an on- or off-target event. Here we investigated the possibility for adverse effect *in vivo* in mice and whether it involves the TRPC4/TRPC5 channels.

## RESULTS

### EA induces adverse reaction

**Figure 1:**
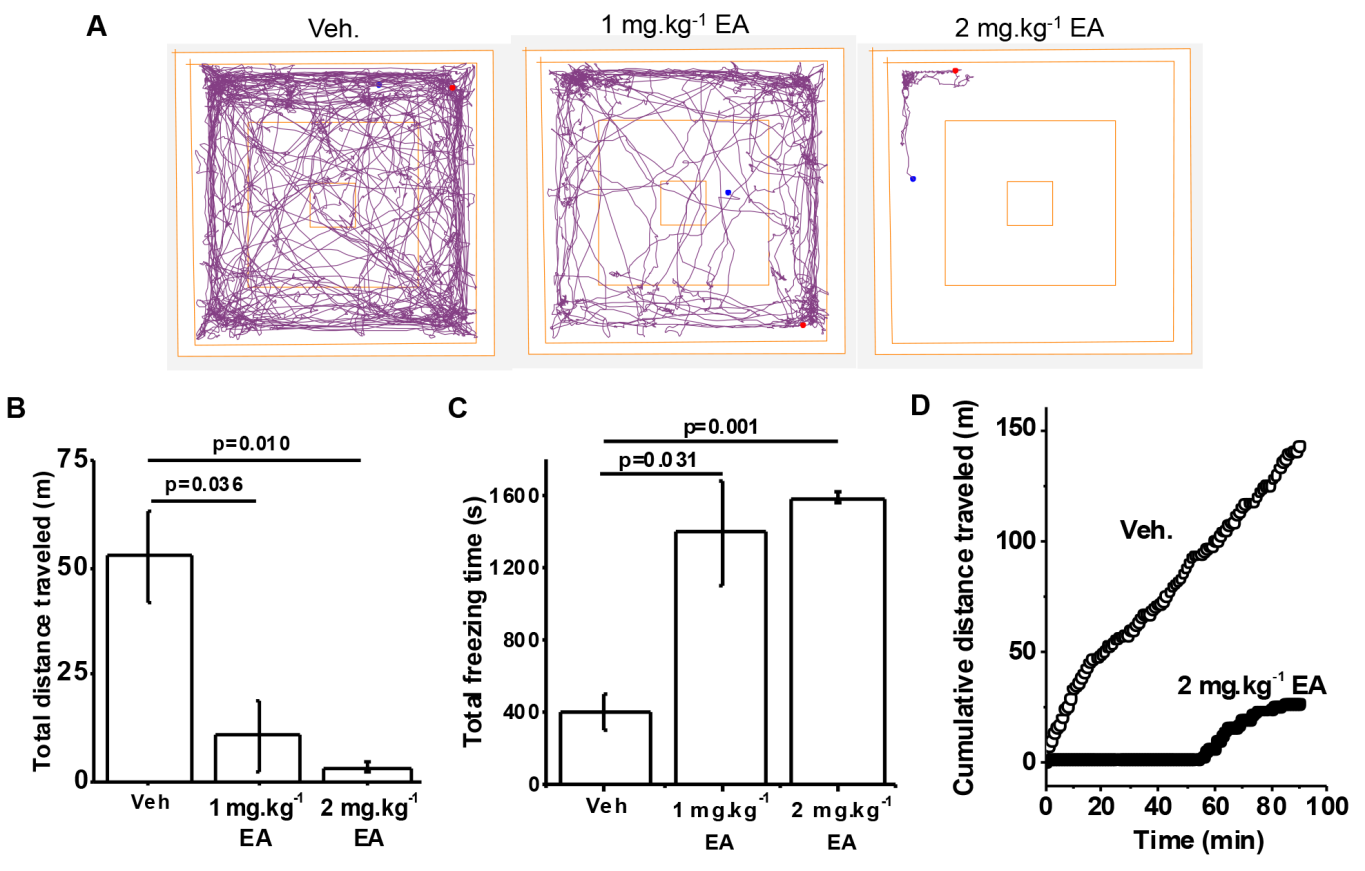
Adverse reaction to (-)-Englerin A (EA) in wild type mice **(A)** Representative track plots generated from the Open Field Test on mice administered with Vehicle (Veh.), 1 mg.kg^-1^ EA or 2 mg.kg^-1^ EA. **(B)** Total distance travelled and, **(C)** total freezing time of mice administered Vehicle (Veh.), 1 mg.kg^-1^ EA or 2 mg.kg^-1^ EA (n=3 per group). **(D)** Examples for individual mice of cumulative distance travelled plotted against time for Vehicle (Veh.) and 2 mg.kg^-1^ EA injection. Representative of n=9 each.

Previous study suggested that intraperitoneal injection of EA in nude mice at >1 mg.kg^-1^ was not tolerated [7]. Using a similar formulation we investigated EA at 1 or 2 mg.kg^-1^ in wild type C57BL/6 mice. A rapid effect on locomotor activity was observed, which we quantified using the Open Field Test (Figure 1A). EA 2 mg.kg^-1^ had a more consistent effect than 1 mg.kg^-1^ but both doses severely reduced the total distance travelled and strongly increased the freezing time (Figure 1A-1C) (Supplementary Video). The Open Field Test illustrated strong negative effect on mice for about 1 hour, followed by recovery (Figure 1D). No mice died at these doses, administered via the intraperitoneal route. Pilot studies suggested that a higher dose of 5 mg.kg^-1^ was poorly tolerated and so further studies at this dose were considered unethical. The data suggest that C57BL/6 mice have negative reaction to EA, which manifests as a transient reduction in locomotor activity.

### Knockout of TRPC4 or TRPC5 partially protects against EA

**Figure 2:**
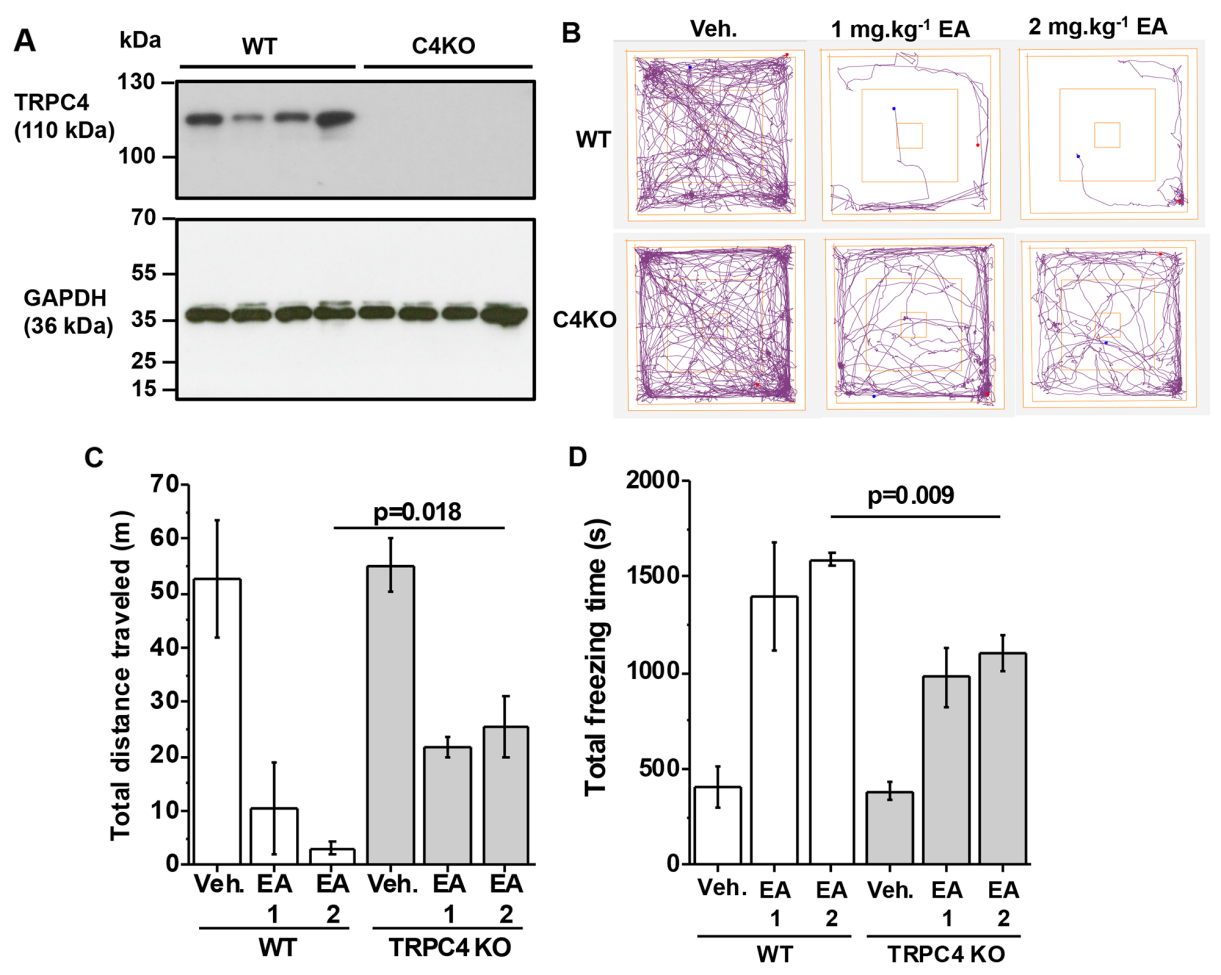
Partial protection by TRPC4 knockout **(A)** Western blot data for total brain proteins from wild type (WT) and TRPC4 knockout (C4KO) mice. Using anti-TRPC4 antibody (upper gel) and anti-GAPDH (lower gel, loading control). Each lane is protein for a different mouse. There were 4 mice in the WT group and 4 mice in the C4KO group. **(B-D)** Data generated for wild type (WT) and TRPC4 knockout mice (TRPC4 KO) in the Open Field Test. (B) Representative track plots, (C) total distance travelled and (D) total freezing time after intraperitoneal injection of vehicle, 1 mg.kg^-1^ EA or 2 mg.kg^-1^ EA (n=3 mice per group).

To test whether TRPC4 is involved in the adverse effect of EA, we next used mice with genetically disrupted *Trpc4* gene. In these mice there was absence of TRPC4 protein (Figure 2A). In vehicle-injected TRPC4 knockout mice (C4KO), distance travelled and freezing times were normal (Figure 2B-2D *cf* Figure 1A-1C). In contrast, the effects of EA on distance travelled and freezing times were significantly attenuated (Figure 2B-2D). The data suggested that TRPC4 knockout partially protected against the adverse effect of EA.

**Figure 3:**
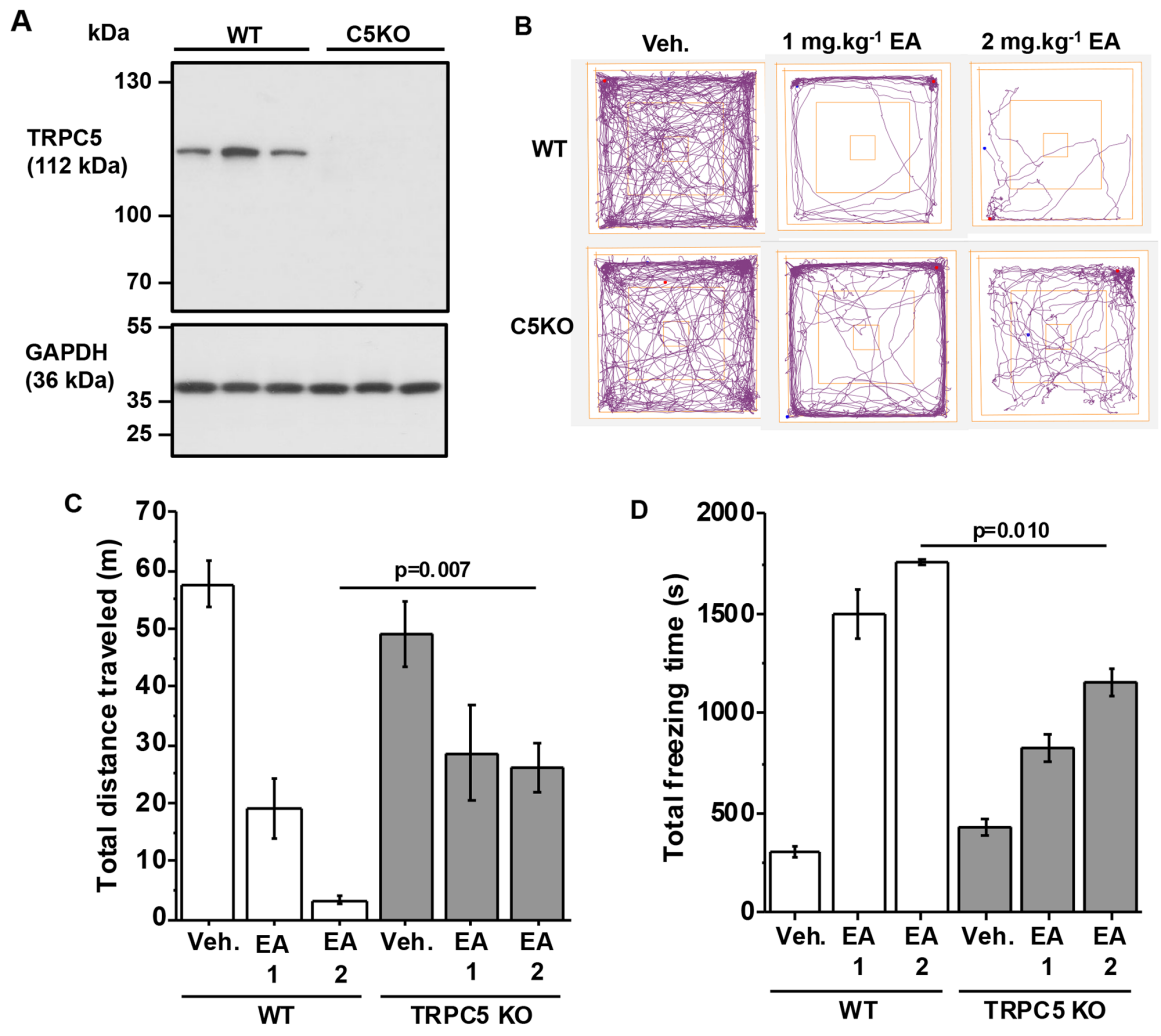
Partial protection by TRPC5 knockout **(A)** Western blot data for total brain proteins from wild type (WT) and TRPC5 knockout (C5KO) mice. Using anti-TRPC5 antibody (upper gel) and anti-GAPDH (lower gel, loading control). Each lane is total protein for a different mouse. There were 3 mice in the WT group and 3 mice in the C5KO group. **(B-D)** Data generated for wild type (WT) and TRPC5 knockout mice (TRPC5 KO) in the Open Field Test. (B) Representative track plots, (C) total distance travelled and (D) total freezing time after intraperitoneal injection of vehicle, 1 mg.kg^-1^ EA or 2 mg.kg^-1^ EA (n=3 mice per group).

We similarly studied TRPC5 knockout mice (C5KO). Absence of TRPC5 protein was confirmed (Figure 3A). As with TRPC4 knockouts, vehicle-injected C5KO mice behaved normally (Figure 3B-3D). In contrast, the effects of EA on distance travelled and freezing times were significantly attenuated (Figure 3B-3D). The data suggested that TRPC5 knockout partially protected against the adverse effect of EA.

The data suggested that TRPC4 and TRPC5 both contributed to the adverse effect of EA. Importantly, substantial adverse effect of EA remained in both knockout mice, suggesting that adverse effect did not depend on TRPC4 or TRPC5 alone.

### Knockout of TRPC4 and TRPC5 fully protects against EA

**Figure 4:**
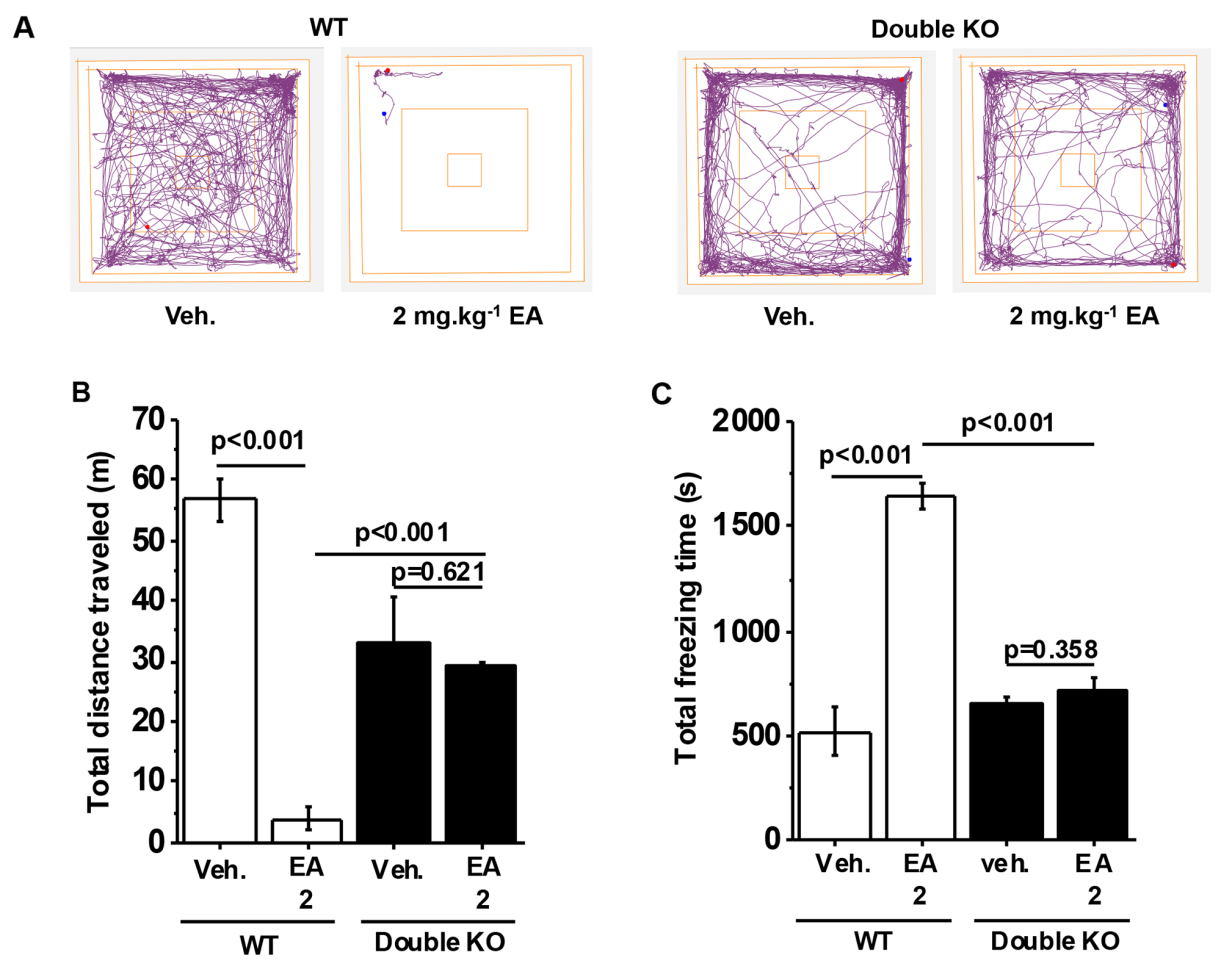
Full protection by TRPC4 and TRPC5 double knockout **(A)** Representative track plots from data generated for wild type (WT) and TRPC4 and TRPC5 double knockout mice (Double KO) in the Open Field Test. **(B)** Total distance travelled and, **(C)** Total freezing time after intraperitoneal injection of vehicle, 1 mg.kg^-1^ EA or 2 mg.kg^-1^ EA (n=3 mice per group).

Because EA activates both TRPC4 and TRPC5 we hypothesized that both proteins mediate a similar adverse effect of EA or that one protein compensates when the other is disrupted. This could explain why the single knockouts only partially protected against EA. Therefore, we generated TRPC4 and TRPC5 double knockout mice (Double KOs). In vehicle-injected Double KOs, the distance travelled was less than in wild type mice but freezing times were similar (Figure 4A-4C). Despite this difference in baseline for distance travelled, it was striking that there was complete protection against EA (Figure 4A-4C). The data suggested that adverse reaction to EA depends completely on a combination of TRPC4 and TRPC5. No adverse reaction was observed in Double KO mice.

Previous study has suggested a particularly severe reaction to intravenous injection of EA [7]. Such an effect might involve a mechanism which is different from that observed in response to intraperitoneal injection. We therefore administered EA intravenously in wild type (WT) mice and TRPC4 and TRPC5 double knockout mice (Double KOs). EA 2 mg.kg^-1^ caused rapid lethality (n=1) and so studies with this dose were discontinued and lower doses were explored. At 0.02 mg.kg^-1^ EA, wild type mice showed mild signs of physiological distress including increased grooming and spontaneous movement within the cage (n=3). In contrast, Double KOs exhibited no response to 0.02 mg.kg^-1^ EA, behaving apparently normally (n=3). At 0.2 mg.kg^-1^ EA there was rapid lethality in wildtype mice, whereas in Double KOs there were signs of palpitation which then subsided within 30 s and mice recovered fully with no obvious symptoms thereafter (n=3). The data suggested that adverse reaction to intravenous EA depends on TRPC4 and/or TRPC5.

### Small-molecule inhibition of TRPC4/TRPC5 channels fully protects against EA

**Figure 5:**
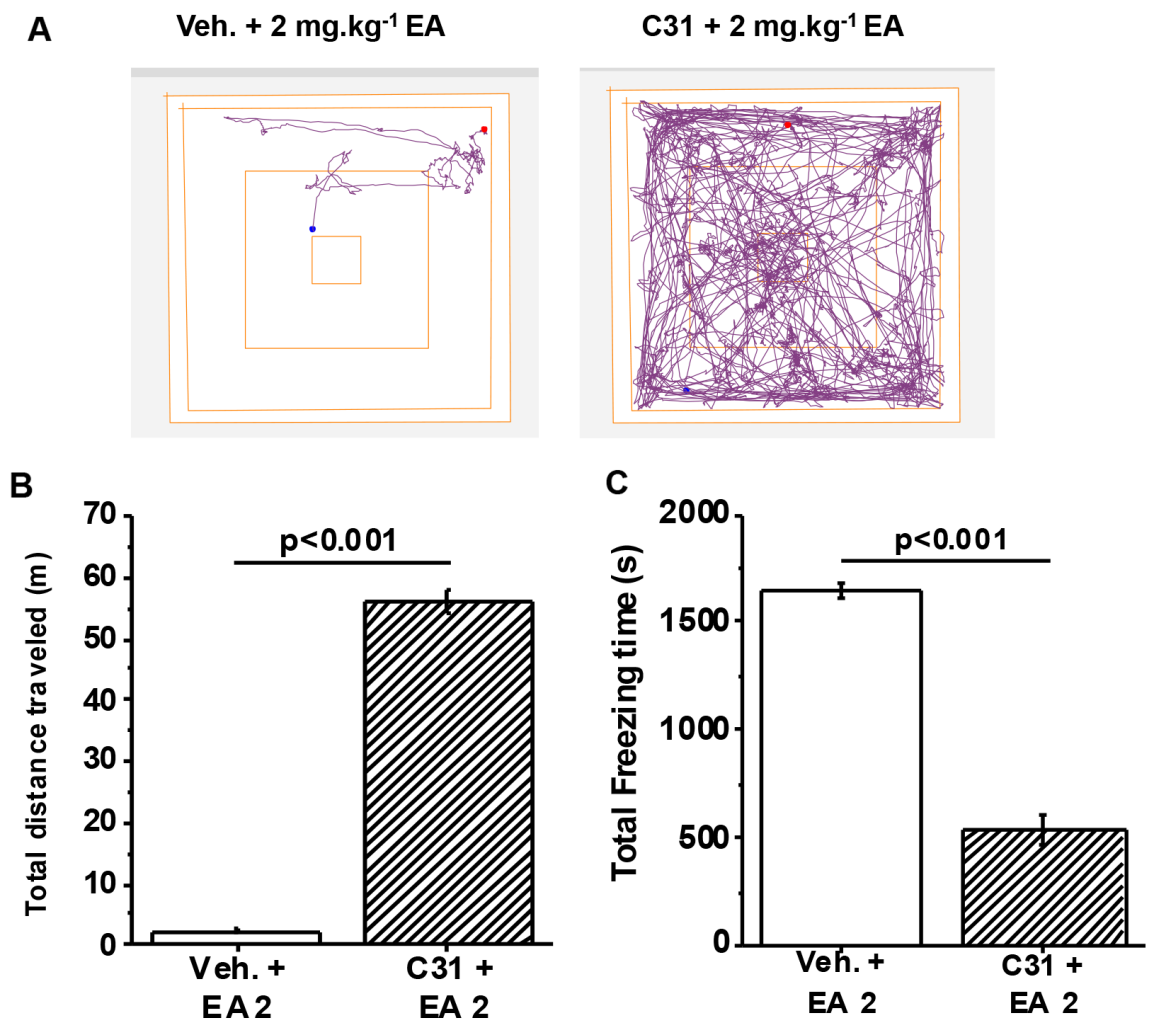
Full protection by a small-molecule inhibitor of TRPC4/TRPC5 channels **(A)** Representative track plots from the Open Field Test using wild type C57BL/6 mice. Mice were orally administered 1 mg.kg^-1^ Compound 31 (C31) or its vehicle 1 hr prior to intraperitoneal injection of 2 mg.kg^-1^ EA. **(B)** Total distance travelled and, **(C)** Total freezing time after intraperitoneal injection of vehicle or EA (n=3 mice per group).

To investigate the role of TRPC4/TRPC5 channels independently of the genetic approach we took advantage of a newly-identified potent and specific inhibitor of these ion channels called Compound 31 (C31), a.k.a. Pico145 [12]. Importantly, pre-administration of C31 fully protected against the adverse effect of EA injected intraperitoneally, such that distance travelled and freezing times were similar to those of mice without EA injection (Figure 5A-5C *cf* Figure 1A-1C). The data supported the hypothesis that TRPC4/TRPC5 channels are essential for adverse response to EA.

### EA is peripherally restricted

**Figure 6:**
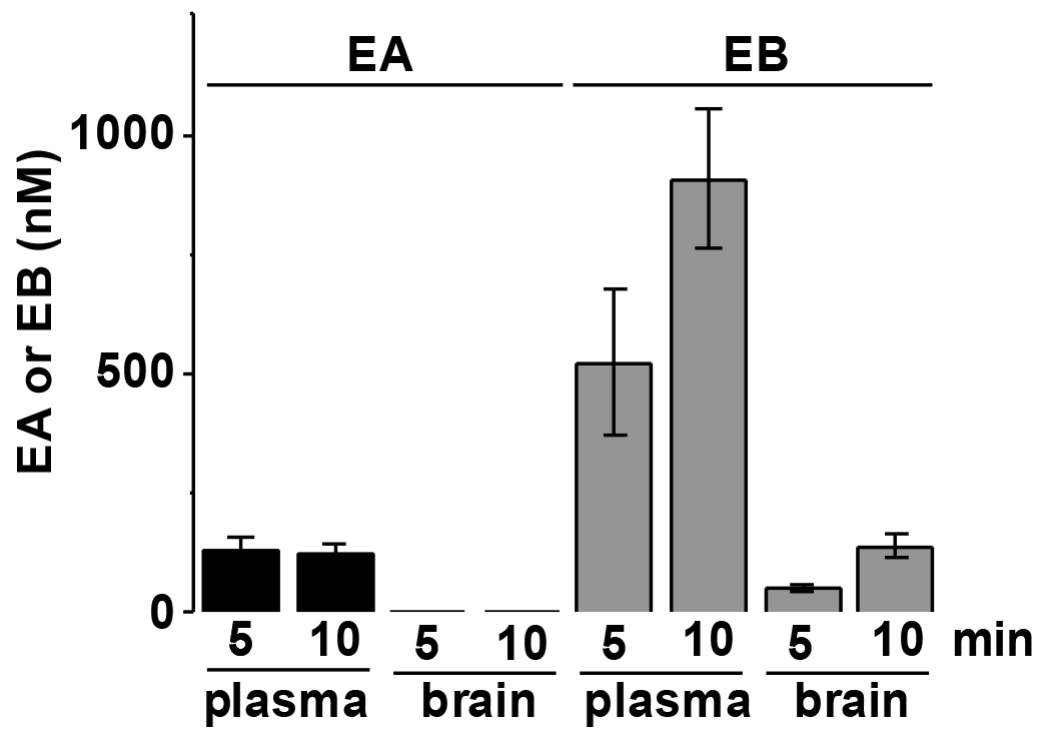
EA distributes peripherally and not to the brain Analysis of the concentration of EA and its metabolite Englerin B (EB) in plasma and brain of nude mice 5 and 10 min after intraperitoneal injection of EA 5 mg.kg^-1^ (n=3 mice). EA was not detected in the brain.

TRPC4 and TRPC5 channels are expressed in the central nervous system where their activation has been associated with epilepsy and fear [13–15]. We therefore investigated whether EA is distributed to the brain. EA 5 mg.kg^-1^ was injected intraperitoneally under anaesthesia in nude mice. Its concentration and that of its metabolite Englerin B (EB) were measured in plasma and brain 5 and 10 min after injection. EA was detectable in plasma but not brain whereas EB was detectable in both, albeit at lower concentration in brain (Figure 6). Intriguingly, EA 5 mg.kg^-1^ was not lethal to these anaesthetized mice. The data suggested the EA is peripherally restricted and that the adverse reaction is likely to be mediated peripherally.

## DISCUSSION

This study supports the existence of adverse reaction to EA in mice and reveals the essential role of the combination of TRPC4 and TRPC5 in the adverse reaction. The reaction is dose-dependent and lethal at high dose. At the dose of 2 mg.kg^-1^ the reaction is severe, manifesting as a significant reduction in locomotor activity, but not lethal, with mice gradually recovering to an apparently normal state. Strikingly, knockout of TRPC4 or TRPC5 and small-molecule blockade of the channels had no obvious effect on their own but knockout of either protein partially protected against this reaction to EA and double knockout, or small-molecule blockade, completely protected against EA. EA did not distribute significantly to the brain but was detected in plasma, suggesting a peripheral mechanism underlying the adverse reaction. The identity of this mechanism is unknown, other than that TRPC4 and TRPC5 are critical.

Anti-cancer cell effects of EA are critically-dependent on TRPC4 [7, 8]. Therefore, our data indicate that the adverse effect of EA is on target (mediated by the same mechanism as the anti-cancer cell effect). Our studies and those of other groups have not supported the idea of EA having a high-affinity target other than TRPC4 and TRPC5 [7, 8]. The suggested effect of EA on Ca_V_1.2 voltage-gated Ca^2+^ channels reflects a low-affinity target in the middle micromolar concentration range [16], which is about 3 orders of magnitude worse than the potency at TRPC4 or TRPC5 channels [7, 8].

The adverse reaction induced by EA was sustained for about 1 hour, after which the mice began to recover. The reason for the transient nature of the effect is likely to be metabolic instability of EA in rodents – probably its conversion to the metabolite Englerin B (EB) which is inactive as an agonist at TRPC4 and TRPC5 channels [7]. Therefore, if a metabolically stable EA analogue can be discovered with retained potent agonistic action at TRPC4 and TRPC5 channels we anticipate that it would cause even more severe adverse reaction.

Our findings add to prior knowledge [17] by showing that Compound 31 (a.k.a. Pico145) is an inhibitor of TRPC4 and TRPC5 channels *in vivo*. On its own, C31 appeared to be without adverse effect, although we only made visual observations of the mice for one hour post-administration before injecting EA. More recently, an anxiolytic and anti-depressant effect in mice of HC-070, a highly similar compound to C31 that also inhibits TRPC4 and TRPC5 channels with high potency and selectivity, has been described [18].

The focus of our study was to examine the role of TRPC4 and TRPC5 channels in mediating adverse reaction to EA. The underlying nature of the adverse reaction to EA *in vivo*, as a consequence of activating TRPC4/5 channels peripherally, remains uncertain but manifests as reduction in locomotor activity. A similar effect was reported after subcutaneous injection of 3 mg.kg^-1^ EA in nude mice [7]. Since the cytotoxic effects of EA are thought to be cancer cell specific, we do not believe direct cytotoxicity to be a likely mechanism but cannot exclude it. Labored breathing, possibly secondary to pulmonary edema, and elevations or occasional reductions of blood pressure, without effect on cardiac contractility, has been suggested after subcutaneous and intravenous injection of EA, respectively, in nude mice [7]. Whilst we did not observe labored breathing in treated mice, it is possible that alterations in vascular or cardiac function were responsible for the observed reaction. Roles of TRPC4 and TRPC5 located to the central nervous system [14, 15] seem unlikely in light of the exclusive peripheral distribution of EA. Skeletal muscle weakness could be an explanation but we noted that the mice remained on their feet and the thorax palpitated, so weak muscle tone is unlikely. Gastrointestinal spasm may contribute because of the important role of TRPC4 channels in contractile function of the intestines [19]. These are but a few possibilities. The broad expression of TRPC4 and TRPC5 will make it challenging to identify the mechanism of EA toxicity. Furthermore, it remains unclear if the mechanism causing the transient reaction observed in our study is directly related to lethality or if there are two mechanistically unrelated effects.

Novel cancer therapeutics are urgently required in the clinic. Renal cell carcinoma, for example, is resistant to traditional cytotoxic chemotherapies and remains incurable in patients with metastatic disease. VEGF-receptor targeted tyrosine kinase inhibitors form a mainstay of current treatment, although on-target side-effects such as diarrhea and hypertension are well recognized. In the majority of patients, such toxicity can be managed safely, with an acceptable risk/benefit ratio achieved. Given that the *in vivo* anti-cancer activity of EA remains to be clearly established, together with the rapidity, severity and, as yet, poorly defined nature of the toxicity associated with EA, significant concerns around the potential of EA analogues as anti-cancer agents in the clinic must remain.

We used the minimum number of animals and did not exceed dosing at 2 mg.kg^-1^ in conscious mice because of ethical constraints. The study was not designed to explore the effects of TRPC4 and TRPC5 knockouts on murine behavior. The data obtained with these knockouts in the absence of EA administration were nevertheless suggestive that neither TRPC4 nor TRPC5 alone was important for the parameters measured by the Open Field Test. However, the absence of both TRPC4 and TRPC5 reduced the times in the center and intermediate zones of the field as well as the total distance travelled.

In conclusion, the study importantly confirms adverse reaction to EA in mice and shows clearly that the mechanism requires both TRPC4 and TRPC5. Because the most common cancer cell cytotoxic effect of EA requires TRPC4 and not TRPC5, the study suggests partial overlap of the potential anti-cancer effect and the adverse reaction of EA. A TRPC4-specific agonist, should it be developed, might therefore be an approach for achieving an anti-cancer effect with tolerable toxicity, but careful dose-dependency studies would be needed with a stable analogue of the agonist before clinical trials could reasonably be considered.

## MATERIALS AND METHODS

### Animals

*Trpc4* gene-disrupted (knockout) mice on the C57BL/6J background (B6.129P2-Trpc4^tm1Dgen^/H) were generated by Deltagen Inc. and supplied by the Medical Research Council Harwell, UK. The sequence spanning base 1272 to base 1330 of the TRPC4 gene was deleted, where it was inserted with a Lac-Z neo cassette to create a detectable mutation in the TRPC4 knockout mice. *Trpc5* gene-disrupted (knockout) C57BL/6 mice were generated as part of the International Mouse Phenotyping Consortium (IMPC); they were based on *Trpc5* gene-targeted ES cells originally created by the Knockout Mouse Project (KOMP) (Trpc5tm1b(KOMP)Wtsi) and provided by Riken BRC, Japan. Mice were intercrossed to generate homozygous single and double knockouts. Mice were weaned at 3 weeks of age and 2-5 mice were housed in the same cage with same-sex littermates under a 12 hour light/dark cycle. Mice of either sex were used. The wild type mice were from the same line of the KO mice for experiments where the effect of EA was being compared between wild type and knock-out mice. For experiments using the C31 compound, the wild type mice were from the TRPC4 line. Wild type and knockout pairs were sex-matched. Food pellets and water were provided *ad libitum*. All procedures were approved by the University of Leeds Animal Welfare and Ethical Review Body and were conducted under a moderate protocol on a project licence issued by the competent authority of the United Kingdom. Englerin distribution studies were carried out at Synovo GmbH Tübingen using female athymic nude mice obtained from Taconic. All experimental procedures were approved by and conducted in accordance with the regulations of the local Animal Welfare Authorities (Tübingen Regional Council).

### Open field test

Experiments were conducted largely as described previously [20]. Animals used were 6-8 weeks of age, sex-matched for wild type and knockout pairs to be compared. Mice were placed into a 40 x 40 x 40 cm arena illuminated under standard white fluorescent ceiling lights at an intensity of ∼200 lux. All experiments were recorded using a webcam attached to a tripod positioned above the arena, connected to the computer tracking software ANY-maze (Stoelting Co., USA). The tracking software divided the arena into three zones: Outer Zone (8 cm from the outer walls), Center Zone (6.4 cm^2^; 16% of the total area) and Intermediate Zone (the remaining area between the outer and center zone). The duration (time pressed, seconds) of rearing and grooming was manually measured.

### Sample preparation for EA and Englerin B (EB)

EA and EB were extracted from plasma and tissue by protein precipitation using acetonitrile. Brain samples were homogenized in PBS prior to extraction. Filtrates were further diluted with mobile phase and analyzed using a Shimadzu Prominence UFLC system coupled to an ABSciex QTrap 5500 instrument. EA and EB were separated by gradient elution using an Agilent Poroshell EC 120 C18 column. Mobile phase A consisted of water containing 10 mM ammonium acetate, and mobile phase B consisted of acetonitrile containing 10 mM ammonium acetate with a flow rate of 1 ml.min^-1^. Ionization of EA and EB was achieved by electrospray ionization (ESI) in positive mode. Mass spectrometer parameters were optimized previously for both analytes. Concentrations of EA and EB were calculated by means of a standard curve using spiked biological sample matrix. Peak processing and curve fitting was performed using Analyst 1.6.2 software from ABSciex.

### Western blotting methods and antibodies

Tissues were homogenized in Radio-Immuno precipitation Assay (RIPA) buffer containing (in mM) 150 NaCl, 20 Tris-base, 1 EGTA, 1 EDTA, 1% NP-40, 0.1% sodium dodecyl sulfate (SDS) and 1% sodium deoxylate, pH 7.6 (HCl). cOmplete™ protease inhibitor (Roche Life Science) was prepared in solution in accordance with the manufacturer’s instructions and added into RIPA before use. Western blotting was carried out under standard protocols with primary antibodies TRPC4 (1:200, 75-119, UC Davis/NIH Neuromab), and TRPC5 (1:200, 75-104, UC Davis/NIH Neuromab). GAPDH (1:4000, AM4300; Ambion Life Technologies) was used as loading control. Subsequent to primary antibody incubation, secondary antibodies anti-rabbit-HRP (1:3000, 711-035-152, Jackson ImmunoResearch) and anti-mouse-HRP (1:3000, 715-035-150, Jackson ImmunoResearch) were used. Chemiluminescence of detected proteins were visualized using SuperSignal® West Femto Maximum Sensitivity Substrate (Thermo Fisher Scientific).

### Chemicals

(-)-Englerin A (EA) was supplied by Phytolab GmbH. The stock solution was made at 10 mg.ml^-1^ in the Novartis approved ‘standard acceptable vehicle guidance for all in-life pre-clinical evaluations’ [7] containing 5% ethanol, 10% polyethylene glycol 300 (Sigma Aldrich), 5% cremophor EL (Merck Chemicals Ltd), and 80% PBS. The stock solution was sonicated for 10 min to dissolve EA and filtered through a 0.22 μm filter prior to further dilution and injection. For oral administration into mice by gavage, Compound 31 was dissolved in 0.5% methylcellulose, viscosity 400 cP (Thermo Fisher Scientific) and PBS. Compound 31 (C31, a.k.a. Pico145) was synthesized as previously described [12]. Buprenorphine Hydrochloride was Buprenex® and Carprofen was Rimadyl® diluted in PBS for subcutaneous injection.

### Data analysis

Data are presented as mean ± SEM. n represents the number of independent experiments (e.g. mice). Comparisons of 2 data groups were by unpaired *t*-test. Multiple groups were compared by ANOVA with post hoc Bonferroni test. Values of statistical differences are provided in the figures. Data were analyzed using Origin software (OriginLab, Northampton, MA, USA).

## SUPPLEMENTARY MATERIALS VIDEO




