# Supplementary material for: TRPC4/TRPC5 channels mediate adverse reaction to the cancer cell cytotoxic agent (-)-Englerin A
Source: Oncotarget. 2018 Jul 3;9(51):29634–43. doi: 10.18632/oncotarget.25659 (PMC6049859; doi:10.18632/oncotarget.25659)
Supplement: Supplementary file 1 [file oncotarget-09-29634-s001.pdf]

TRPC4/TRPC5 channels mediate adverse reaction to the cancer cell cytotoxic agent (-)-Englerin A

SUPPLEMENTARY MATERIALS

Supplementary Video 1: Video showing effect on wild type C57BL/6 mouse immediately following intraperitoneal injection of (-)-Englerin A 2mg.kg^{-1}.

See Supplementary Video 1
